# Supplementary material for: Research-based occupational therapy education: An exploration of students’ and faculty members’ experiences and perceptions
Source: PLoS One. 2020 Dec 21;15(12):e0243544. doi: 10.1371/journal.pone.0243544 (PMC7751851; doi:10.1371/journal.pone.0243544)
Supplement: S1 File — (DOCX) [file pone.0243544.s001.docx]

| **Interview guide for faculty members** |
| --- |
| **INTRODUCTION**  Norwegian higher education institutions are required by law to base their programmes on state-of-the-art research, and a recent white paper from the Ministry of Education and Research (Kunnskapsdepartementet 2016) underscores the need for research-based education. In this interview, we will focus on your experience with the use of research in education and training.  We would like to find out how you perceive and experience the emphasis on research in the study programme, and how you perceive the requirements for students to use research in academic programmes and clinical placements. |
| **INTRODUCTORY QUESTION**  *How do you interpret the concept ‘research-based education’?* |
| **QUESTIONS BY TOPIC**   1. **Research-based education**   Can you describe how research is applied to your study programme? For example in the teaching, supervision and generally in the encounter with students?   - In what way is the occupational therapy study programme research-based? - Do you refer to your own research? Please describe situations where you have used/referred to your own or others’ research in teaching, supervision etc. - Advantages and disadvantages of the emphasis on research in the occupational therapy study programme? - Are you affiliated with a research community?  1. **Requirements for the students’ use of research-based knowledge**   How do you perceive the requirements for students when it comes to using research in their study programme?   - What are these requirements? Any differences between (academic) on-campus activities and clinical placements? - Do you notice any progression over the three years – could you describe?  1. **Participation in R&D projects**   Could you give any examples of students being involved in research projects during their study programme?   - Any advantages or disadvantages? - What do they learn from this?  1. **Future professional practice**   In your opinion, what effect could research-based education have on the candidates’ future professional practice?   - Exposure/training/competence  1. **Educational research**   Are you familiar with, or have you participated in research projects on study programmes, teaching and student learning? |
| **CONCLUSION:** Before we end, do you have something you wish to add? |
