# Supplementary material for: Research-based occupational therapy education: An exploration of students’ and faculty members’ experiences and perceptions
Source: PLoS One. 2020 Dec 21;15(12):e0243544. doi: 10.1371/journal.pone.0243544 (PMC7751851; doi:10.1371/journal.pone.0243544)
Supplement: S2 File — (DOCX) [file pone.0243544.s002.docx]

| **Intervjuguide til vitenskapelig ansatte** |
| --- |
| **INTRODUKSJON**  I norsk høyere utdanning er det et lovpålagt krav at alle utdanninger er basert på det fremste innen bla forskning og i ny kvalitetsmelding fra kunnskapsdepartementet (kunnskapsdepartementet 2016) fremheves behovet for forskningsbasert utdanning. Fokuset for denne samtalen er dine/deres erfaringer med bruk av forskning i utdanningen.  Vi er interessert i å finne ut hvordan dere opplever og erfarer at forskning blir vektlagt i utdanningen, og hvordan dere opplever krav som stilles til studenter når det gjelder bruk av forskning i akademiske emner og i praksisperioder. |
| **INNLEDENDE SPØRSMÅL**  *Hvordan forstår dere begrepet forskningsbasert utdanning?* |
| **TEMASPØRSMÅL**   1. **Forskningsbasert utdanning**   Kan dere beskrive hvordan forskning anvendes i utdanningen hos dere? For eksempel i undervisning, veiledning og generelt i møte med studenter?   - På hvilken måte er ergoterapiutdanningen forskningsbasert? - Henvises det til egen forskning? Beskriv situasjoner der dere har brukt/henvist til egen/andres forskning i undervisning, veiledning o.l? - Fordeler og ulemper med at forskning vektlegges i ergoterapiutdanningen? - Er dere tilknyttet et forskningsmiljø?  1. **Krav til studenters bruk av forskningsbasert kunnskap**   Hvordan opplever dere krav som stilles til studenter når det gjelder å anvende forskning i utdanningen?   - Hvilke krav stilles? Forskjeller i emner som foregår på campus (akademisk) og praksisperioder? - Opplever dere at det er en progresjon gjennom de tre årene – kan dere beskrive?  1. **Deltakelse i FOU prosjekt**   Kan dere gi eksempler på at studenter er involvert i forskningsprosjekter ved utdanningen?   - Fordeler og ulemper? - Hva lærer de av dette?  1. **Fremtidig profesjonsutøvelse**   Hvilken betydning tror dere at forskningsbasert utdanning kan ha for fremtidig profesjonsutøvelse?   - Eksponering/opplæring/kompetanse  1. **Utdanningsforskning**   Kjenner du til eller har du deltatt i forskningsprosjekter i forhold til studieprogrammene, undervisning og studentenes læring? |
| **AVSLUTNING:** Avslutningsvis, har dere noe dere vil tilføye? |
