# Supplementary material for: Research-based occupational therapy education: An exploration of students’ and faculty members’ experiences and perceptions
Source: PLoS One. 2020 Dec 21;15(12):e0243544. doi: 10.1371/journal.pone.0243544 (PMC7751851; doi:10.1371/journal.pone.0243544)
Supplement: S3 File — (DOCX) [file pone.0243544.s003.docx]

***HOW DO OCCUPATIONAL THERAPY STUDENTS AND FACULTY MEMBERS PERCEIVE THE EMPHASIS ON RESEARCH IN THE BACHELOR’S DEGREE PROGRAMME?***

| **Interview guide for students** |
| --- |
| **INTRODUCTION** Norwegian higher education institutions are required by law (Universities and University Colleges Act) to base their programmes on state-of-the-art research, and a recent white paper from the Ministry of Education and Research (Kunnskapsdepartementet 2016) underscores the need for research-based education. The objective of this project is to improve our knowledge on research-based education in occupational therapy. This interview will focus on the students’ viewpoints and experiences regarding research-based knowledge in the study programme. This could include the students’ perceptions of how research-based knowledge is emphasised and the requirements for use of research in the study programme. |
| **INTRODUCTORY QUESTION**  *How do you interpret the concept ‘research-based education’? What do you think of as ‘research-based knowledge`?* |
| **QUESTIONS BY TOPIC**   1. **Research-based education/research-based knowledge**   *Could you describe one or more situations where you have used research-based knowledge during your study programme?*   - What is your experience of reading and using research literature in your studies? - Can you describe situations where research literature was used in the training programme? What are the advantages of this? - How do you perceive the requirements that are placed on students when it comes to using research-based knowledge in their studies? - Do you have the impression that the teachers themselves engage in research? - Could you describe how your teachers make use of and communicate research results in their teaching and supervision? What are the advantages of this?  1. **Research methods and philosophy of science**  - How do you perceive the teaching and supervision in research methodology and philosophy of science in the training programme? What advantages do you see in emphasising these topics?  1. **Participation in R&D projects**  - Have you participated in a research project or research-related activities during your studies? Could you describe your experiences in this regard?  1. **Clinical placements**   *Could you describe one or more situations where you have used research-based knowledge during clinical placements/in the encounter with patients?*   - Could you describe situations where you saw that your supervisor used research-based knowledge during your clinical placements? - Could you describe the requirements that you perceive to be placed on you when it comes to using research-based knowledge in clinical placements? On campus and in clinical placements. Please use examples.  1. **Future professional practice**  - How important do you think research-based knowledge will be for your role as a professional in the future? |
| **CONCLUSION:** Before we end, do you have something you wish to add? |
