# Supplementary material for: Research-based occupational therapy education: An exploration of students’ and faculty members’ experiences and perceptions
Source: PLoS One. 2020 Dec 21;15(12):e0243544. doi: 10.1371/journal.pone.0243544 (PMC7751851; doi:10.1371/journal.pone.0243544)
Supplement: S4 File — (DOCX) [file pone.0243544.s004.docx]

***HVORDAN ERFARER ERGOTERAPISTUDENTER OG VITENSKAPELIG ANSATTE FORSKNINGSBASERT UTDANNING VED BACHELORPROGRAM I ERGOTERAPI?***

| **Intervjuguide til studentene** |
| --- |
| **INTRODUKSJON** I norsk høyere utdanning er det et lovpålagt krav (universitets- og høgskoleloven 2005) at alle utdanninger er basert på det fremste innen blant annet forskning og i ny kvalitetsmelding fra kunnskapsdepartementet (kunnskapsdepartementet 2016) fremheves behovet for forskningsbasert utdanning. I dette prosjektet er målet å styrke kunnskapen om forskningsbasert utdanning knyttet til ergoterapiutdanningen. Fokuset for denne samtalen er studenters synspunkter og erfaringer med forskningsbasert kunnskap i utdanningen. Det kan være hvordan studenter opplever at forskningsbasert kunnskap blir vektlagt i utdanningen, og hvordan studenter opplever krav som stilles når det gjelder bruk av forskning i utdanningen. |
| **INNLEDENDE SPØRSMÅL**  *Hvordan forstår dere begrepet forskningsbasert utdanning? Hva legger dere i forskningsbasert kunnskap?* |
| **TEMASPØRSMÅL**   1. **Forskningsbasert utdanning/forskningsbasert kunnskap**   *Kan dere beskrive en eller flere situasjoner der du/dere har brukt forskningsbasert kunnskap i utdanningen?*   - Hvilken erfaring har dere med å lese og anvende forskningslitteratur gjennom utdanningen? - Kan dere beskrive situasjoner der forskningslitteratur har blitt brukt i utdanningen? Hvilke fordeler gir dette? - Hvordan opplever dere kravene som stilles til dere som studenter når det gjelder bruk av forskningsbasert kunnskap i utdanningen? - Har dere inntrykk av at lærerne selv driver med forskning?   Kan dere beskrive hvordan lærerne ved utdanningen bygger på og formidler forskningsresultater i undervisning og veiledning? Hvilke fordeler gir dette?   1. **Forskningsmetode og vitenskapsteori**  - Hvordan har dere erfart undervisning og veiledning om forskningsmetode og vitenskapsteori gjennom utdanningen? Hvilke fordeler har dere erfart ved vektlegging av disse temaene?  1. **Deltakelse i FOU-arbeid**  - Har dere deltatt i forskningsprosjekt eller forskningslignende oppgaver i løpet av utdanningen? Kan dere beskrive erfaringer knyttet til dette?  1. **Praksisperioder**   *Kan dere beskrive en eller flere situasjoner der dere har brukt forskningsbasert kunnskap i løpet av praksisperioder/ i møte med pasienter?*   - Kan dere beskrive situasjoner der dere har opplevd at veileder har brukt forskningsbasert kunnskap i praksisperiodene dere har hatt? - Kan dere beskrive hvilke krav dere opplever stilles til dere som studenter når det gjelder bruk av forskningsbasert kunnskap i praksis? Fra utdanning og praksisstedet. Beskriv gjerne med eksempler.  1. **Fremtidig yrkesutøvelse**  - Hvilken betydning tror dere at forskningsbasert kunnskap kan ha for deres rolle som yrkesutøver i fremtiden? |
| **AVSLUTNING** Avslutningsvis, har dere noe dere vil tilføye? |
