# Supplementary material for: Research-based occupational therapy education: An exploration of students’ and faculty members’ experiences and perceptions
Source: PLoS One. 2020 Dec 21;15(12):e0243544. doi: 10.1371/journal.pone.0243544 (PMC7751851; doi:10.1371/journal.pone.0243544)
Supplement: Attachment — Submitted filename: PLOSOne review 07 2020.docx [file pone.0243544.s006.docx]

**Review of:** Research-based occupational therapy education: an exploration of students` and faculty members` experiences and perceptions

**General opinion**

-Exploring the perceptions of OT students and faculty regarding their experiences with receiving education about research is an important topic related to encouraging evidence-based practice in the field of OT. However, after reading your introduction, especially lines 75-107, I believe the concept of “exploring student and faculty attitudes toward research” is too broad a description; the concept of “research” is has many components, and I want to know *what* aspects of “research” the students and faculty are responding to. Generally, I think you are describing implementing curriculum about research *methods* and *practice* into the OT curriculum, as when you mention implementing EBP and RU skills in line 93-95, and these are tangible aspects of a “research” education help clarify the broad topic. In addition, as a qualitative paper, I would expect much more quotes in the results section to support your findings. Instead of merely summarizing the words of the participants, you should considering *using* the words of the participants and honoring the qualitative process. Overall, your paper would benefit by clearly describing what aspects or elements from a research based curriculum the students are responding to – learning research methods, exploring outcomes from interventions, etc., and adding more participant quotes to support your findings.

**Abstract and introduction**

-Introduction Line 54 missing “The” in front of name of WFOT organization

-Are lines 59-69 still from the WFOT guidelines? It’s unclear if you are still referring back to them or not; a transition sentence explaining this as the source for your paginated citations is necessary for clarification

-Line 137 – what is ECTS? You do not describe the acronym before using it

-Line 137 also missing an article “The” Bachelor degree in OT

-Minor issues with grammar, conjugation of verbs, and missing/incorrect prepositions

**Figures and tables**

-I think the figure is not a crucial addition to your manuscript, as it is just a visual representation of your themes. I’d suggest cutting it

**Methods**

-Table 2 is listed before Table 1

-In the Data Collection section, more information is needed regarding the characteristics of the interviewers/those collecting the data (were you also faculty members? Students? How did you achieve buy in?), and who developed the interview guide (a collaborative group of researchers? Did you have a qualitative expert review it? Did you pilot it with a small sample prior to using it, or receive any feedback from member checking?)

-Who are you citing in line 212?

-During the analysis, what strategies did the coders use to address their own biases? More information is needed on this to ensure rigor in the analysis process

**Results, discussion, conclusions**

-Generally, I think all of your themes would benefit from including more direct supporting quotes from participants. My general rule of thumb is 1-2 quotes per concept (depending on length of paper, how many themes presented, etc); in some cases, you merely describe a concept but do not include a quote to support It (like lines 264-282)

-Line 227, 416, and other instances: you mention “interviews”, however, in your methods, you describe conducting focus groups sessions, which are not entirely the same thing as a standard interview. I suggest using terminology reflective of your actual methods, ie changing these instances to say “across all focus groups”

-Line 231 is another example of the vagueness of the idea of “research education”. In your paper, you say “Most students questioned the timing of introducing research to students during their training,” and to read the accompanying quote supporting this concept, you can see that the student clearly mentions *research skills like searching for articles*. The example from the student is the concrete aspect that I am looking for when I say to be more clear in your descriptions of the *what* in research education programs throughout your paper.

-I think you should split line 278 into a new paragraph, and you need a quote to illustrate this dilemma described by the faculty.

-Reduce the redundancy in lines 325-328

-Line 357 cuts off, but it feels as though the rest of the quote might be relevant. Consider editing to add content

-Lines 454-470 are nicely presented, as is most of your discussion, comparing your findings to other findings that support and expand the data

-I would call line 561 “Limitations”

**Writing quality & clarity**

-Please review English language grammar and use of punctuation and missing/incorrect prepositions, as well as verb conjugations

-Review use of commas (lines 173, 210, 228, 461, 544, 580)

**Reference list**

-Remove the hyperlinks
