# Supplementary material for: Research-based occupational therapy education: An exploration of students’ and faculty members’ experiences and perceptions
Source: PLoS One. 2020 Dec 21;15(12):e0243544. doi: 10.1371/journal.pone.0243544 (PMC7751851; doi:10.1371/journal.pone.0243544)
Supplement: Attachment — Submitted filename: Response to Reviewers.docx [file pone.0243544.s007.docx]

**Authors response to reviewers**

PONE-D-20-09978

**Title:** Research-based occupational therapy education: an exploration of students` and faculty members` experiences and perceptions.

**Dear Sara Rubinelli, dear reviewers,**

Thank you for the opportunity to submit a revised version of our manuscript. We appreciate the constructive criticism and helpful feedback from the referees. All reviewers’ comments are addressed in detail below, in the order of the reviewers’ remarks and comments.

**Editor:**

Journal Requirements:

When submitting your revision, we need you to address these additional requirements.

1. Please ensure that your manuscript meets PLOS ONE's style requirements, including those for file naming. The PLOS ONE style templates can be found at

<https://journals.plos.org/plosone/s/file?id=wjVg/PLOSOne_formatting_sample_main_body.pdf> and

<https://journals.plos.org/plosone/s/file?id=ba62/PLOSOne_formatting_sample_title_authors_affiliations.pdf>

Authors: We hope this is taken care of and changed as required.

2. Please include a copy of Table 2 which you refer to in your text on page 14

Authors: We are sorry for this mistake; the manuscript contains only one table (page 9-10, line 212).

Reviewer #1: I have some concern with the way that ‘research-based education’ is conceptualised in the manuscript. Research-based teaching refers to teaching practice “…when academics plan, deliver and assess students’ work through their involvement in research or inquiry-based activities” (p.728, Huet, 2018 – Research-based education as a model to change the teaching and learning environment in STEM disciplines, published in the European Journal of Engineering Education, 43:5, 725-740). A similar term – ‘research-lead teaching’ – occurs when academics use their expertise as active researcher or use the research of others to inform teaching.

Authors: We have described research-based education from an international perspective (page 3-4, line 62-73). The research-based education model by Huet 2018 felt to be appropriate in this manner. The description of research-based education as described by the Norwegian white paper is now in the context section (page 6, line 135-141).

Reviewer #1: Without knowing how the occupational therapy curriculum is structured, I find it difficult to accept that it is indeed research based. It would have been beneficial for the authors to give more detail about how the curriculum is designed and delivered and to provide a few examples of research-based learning activities.

Authors: We have added some examples of research-based learning activities in the context section in the paper (page 7, line 149-158). Curriculum documents in occupational therapy education indicate that they do contain elements of research-based education, yet more elements of research-led teaching than research-based teaching.

Reviewer #1: Why is occupational therapy education considered as a short professional education programme, given that it is a bachelor degree?

Authors: We have changed this to bachelor in health care education throughout the manuscript.

Reviewer #1: What are the expectations placed on the lecturers teaching in the occupational therapy programme, in terms of their engagement with research? Are they all clinicians? Again, providing more information about the course structure might help reduce the confusion here.

Authors: We have added more information regarding the faculty members’ competence and their available time for engagement in research and development in the context section of the paper (page 7, line 159-167).

Reviewer #1: I would have liked to see more rationale given to using interpretive description as the study methodology. What’s the advance of this approach and why is it most suitable for the current study?

Authors: We have added more text regarding interpretive description in the methods section, (page 8, line 174-176). The nature of interpretive description helped us ensure that we were interpreting the data instead of just thematizing it. Interpretive description is also suitable for studying phenomena in applied disciplines such as education.

Reviewer #1: Participants commented on the fear that “… the increased demand for research-based education could cause a distance to professional practice”. It would be important to discuss whether comments like this fits the overall research-based model and to provide solutions to reduce such fears. I personally think that research and teaching should not be competing against each other and that the whole premise of research-based education is to incorporate research into teaching, as a means to facilitate active inquiry and critical thinking.

Authors: This is an important point which we have tried to make clearer. It is highlighted in the discussion section (page 24, line 533-540). A suggested solution is described in the discussion section (page 26-27, line 597-609). We have also added one quote in the results section to describe the participants concern regarding this issue (page 15, line 333-337).

Reviewer #2: 2). The method should be improved to give details of the research methodology to understand more clearly how to do, what to do and how to analyze in order to get reliable research results Due to a small sample number.

Authors: The analysis is described on page 10-11, line 222-239. Issues of rigor and credibility are an important consideration in the creation of an interpretive description study. We have added more information regarding rigor and credibility in our study on page 11, line 239-244, which we hope is clarifying. It is important to reflect upon sample size, and we considered this when conducting our focus groups. We have added text about sample size in the limitations section (page 28, line 636-641).

Reviewer #2: 3) Summary of themes is good but should make the diagram interesting In order for readers to understand the elements discovered in quality research more clearly, will make this research suitable for dissemination that useful in occupational therapy for the publication of world-renowned journals. Should adjust the diagram to be clear.

Authors: The diagram or figure in the results section is now removed. Instead, we have added text to describe the results (page 12, line 251-261).

Reviewer #3 **General opinion**

Reviewer #3 - After reading your introduction, especially lines 75-107, I believe the concept of “exploring student and faculty attitudes toward research” is too broad a description; the concept of “research” is has many components, and I want to know *what* aspects of “research” the students and faculty are responding to. Generally, I think you are describing implementing curriculum about research *methods* and *practice* into the OT curriculum, as when you mention implementing EBP and RU skills in line 93-95, and these are tangible aspects of a “research” education help clarify the broad topic.

Authors: Research-based education is a term that includes many elements. We have tried to describe in more detail how research-based education was explored in our study through our interview guide (page 9, line 200-206).

Reviewer #3: In addition, as a qualitative paper, I would expect much more quotes in the results section to support your findings. Instead of merely summarizing the words of the participants, you should considering *using* the words of the participants and honoring the qualitative process.

Authors: There was only one quote under the theme “setting higher expectations”. We have now added two more quotes to this section (page 15, line 333-337) and (page 16, line 346-350). The other themes had two or more quotes, in line with the “rule of thumb” of including at least two quotes under each theme.

Reviewer #3: Overall, your paper would benefit by clearly describing what aspects or elements from a research based curriculum the students are responding to – learning research methods, exploring outcomes from interventions, etc., and adding more participant quotes to support your findings.

Authors: We have added some examples of research-based learning activities under context (page 7, line 149-158). More participant quotes have been added.

Reviewer #3 **Abstract and introduction**

Reviewer #3 -Introduction Line 54 missing “The” in front of name of WFOT organization

Authors: Changed as required (page 3, line 58).

Reviewer #3 -Are lines 59-69 still from the WFOT guidelines? It’s unclear if you are still referring back to them or not; a transition sentence explaining this as the source for your paginated citations is necessary for clarification

Authors: This is from another reference and has been moved to the context section (page 6, line 135-141).

Reviewer #3 -Line 137 – what is ECTS? You do not describe the acronym before using it

Authors: Changed as required (page 6, line 143-144).

Reviewer #3: -Line 137 also missing an article “The” Bachelor degree in OT

Authors: Changed as required (page 6, line 143).

Reviewer #3 -Minor issues with grammar, conjugation of verbs, and missing/incorrect prepositions

Authors: Large parts of the manuscript have been rewritten, and the manuscript has undergone a second round of proof-reading by a professional service prior to submission.

**Figures and tables**

Reviewer #3 -I think the figure is not a crucial addition to your manuscript, as it is just a visual representation of your themes. I’d suggest cutting it.

Authors: The figure in the results section is now removed. Instead, we have added text to describe the results on page 12, line 251-261.

**Methods**

Reviewer #3: -Table 2 is listed before Table 1

Authors: Changed as required. The manuscript has only one table.

Reviewer #3: -In the Data Collection section, more information is needed regarding the characteristics of the interviewers/those collecting the data (were you also faculty members? Students? How did you achieve buy in?) and who developed the interview guide (a collaborative group of researchers? Did you have a qualitative expert review it? Did you pilot it with a small sample prior to using it, or receive any feedback from member checking?)

Authors: More information about the interviewers is now presented in the data collection section (page 9, line 208-211). Information about how we achieved buy in is included in the methods section (page 8, line 179-183). We have also added information regarding the development of the interview guide (page 9, line 206-208) and regarding the use of member checking in the limitations section (page 28, line 631-634).

Reviewer #3 -Who are you citing in line 212?

Authors: The quotation marks have been removed since we are not citing anyone.

Reviewer #3 -During the analysis, what strategies did the coders use to address their own biases? More information is needed on this to ensure rigor in the analysis process.

Authors: We have added more information regarding rigor and reliability in our study on page 11, line 239-244, which we hope is clarifying.

**Results, discussion, conclusions**

Reviewer #3: -Generally, I think all of your themes would benefit from including more direct supporting quotes from participants. My general rule of thumb is 1-2 quotes per concept (depending on length of paper, how many themes presented, etc); in some cases, you merely describe a concept but do not include a quote to support It (like lines 264-282)

Authors: The theme “setting higher expectations” only had one quote, so we have added two more quotes in that section (page 15, line 333-337) and (page 16, line 346-350). The other themes had two or more quotes, in line with the “rule of thumb” of including at least two quotes under each theme.

Reviewer #3: -Line 227, 416, and other instances: you mention “interviews”, however, in your methods, you describe conducting focus groups sessions, which are not entirely the same thing as a standard interview. I suggest using terminology reflective of your actual methods, ie changing these instances to say “across all focus groups”.

Authors: Changed as required throughout the manuscript.

Reviewer #3: -Line 231 is another example of the vagueness of the idea of “research education”. In your paper, you say “Most students questioned the timing of introducing research to students during their training,” and to read the accompanying quote supporting this concept, you can see that the student clearly mentions *research skills like searching for articles*. The example from the student is the concrete aspect that I am looking for when I say to be more clear in your descriptions of the *what* in research education programs throughout your paper.

Authors: We have tried to be more precise in our description of research-based education, both from an international perspective (page 3, line 62-73), how it can be understood in a Norwegian context (page 6, line 135-141) and how we have explored it in our study through the interview guide (page 9, line 200-206).

Reviewer #3: -I think you should split line 278 into a new paragraph, and you need a quote to illustrate this dilemma described by the faculty.

Authors: This has been changed as required and we have added one quote describing this dilemma (page 15, line 333-337).

Reviewer #3: -Reduce the redundancy in lines 325-328

Authors: Changed as required.

Reviewer #3: -Line 357 cuts off, but it feels as though the rest of the quote might be relevant. Consider editing to add content

Authors: one word was missing. This is changed as required, see page 18 line 402.

Reviewer #3: -I would call line 561 “Limitations”

Authors: Changed as required.

**Writing quality & clarity**

Reviewer #3: -Please review English language grammar and use of punctuation and missing/incorrect prepositions, as well as verb conjugations.

Authors: Large parts of the manuscript have been rewritten, and the manuscript has undergone a second round of proof-reading by a professional service prior to submission.

Reviewer #3: -Review use of commas (lines 173, 210, 228, 461, 544, 580)

Authors: Large parts of the manuscript have been rewritten, and the manuscript has undergone a second round of proof-reading by a professional service prior to submission.

**Reference list**

Reviewer #3: -Remove the hyperlinks

Authors: changed as required.
